# Comparison of the Effectiveness of Vojta Therapy and the NDT Bobath Concept in the Treatment of Congenital Muscular Torticollis in Infants—A Retrospective Cohort Pilot Study

**DOI:** 10.3390/jcm15031286

**Published:** 2026-02-05

**Authors:** Marcin Machnia, Adam Płusajski, Ewelina Leśniak, Karolina Urazińska, Wojciech Kałużyński

**Affiliations:** Department of Rehabilitation, Polish Mother’s Memorial Hospital—Research Institute in Lodz, 93-338 Łódź, Poland; adam.plusajski@iczmp.edu.pl (A.P.); ewelina.lesniak@iczmp.edu.pl (E.L.); karolina.urazinska@iczmp.edu.pl (K.U.); wojciech.kaluzynski@iczmp.edu.pl (W.K.)

**Keywords:** congenital muscular torticollis, Vojta therapy, NDT Bobath concept, infant physiotherapy, cervical mobility, early intervention

## Abstract

**Background/Objectives**: Congenital muscular torticollis (CMT) affects 0.3–3.9% of infants, requiring early physiotherapy to prevent deformities. Vojta and NDT Bobath therapies are widely used, yet comparative evidence remains limited. To compare Vojta versus NDT Bobath efficacy in improving head tilt and cervical rotation in infants with CMT. **Methods**: Retrospective cohort study (2016–2024) at Polish Mother’s Memorial Hospital included 53 infants under 5 months with ultrasound-confirmed CMT. Non-random allocation based on therapist availability introduced selection bias. Participants received Vojta (*n* = 29) or NDT Bobath (*n* = 24) two 30 min sessions weekly for 20 weeks plus home exercises. Blinded physicians measured outcomes. **Results**: Vojta showed greater angular improvements versus NDT Bobath: head tilt MD = −5.69° (*p* < 0.001, Hedges’ g = 1.29) and neck rotation MD = −5.89° (*p* < 0.001, Hedges’ g = 1.21). Early intervention (1–2 months) demonstrated 5-fold (RR = 5.46) and 8-fold (RR = 8.19) higher likelihood of achieving optimal thresholds (70°/90°) versus later intervention (3–4 months) both *p* < 0.001. No therapy × age interaction was found, indicating consistent between-group differences across age strata. Large effect sizes suggest clinically meaningful angular improvements. **Conclusions**: Vojta therapy was associated with superior angular outcomes versus NDT Bobath, with early initiation showing better results. However, the retrospective non-randomized design, small sample (*n* = 53), and absence of functional outcome assessment limit causal inference. Only biomechanical outcomes were measured; functional motor development, complications, and quality of life were not evaluated. Prospective randomized trials with functional assessments and larger samples are essential to confirm these associations and determine clinical significance.

## 1. Introduction

The term torticollis originates from Latin: “tortus”—twisted or tilted, and “collum”—neck. It refers to involuntary head tilt and neck rotation due to muscle or cervical spine changes. Congenital muscular torticollis is the third most common congenital musculoskeletal disorder in infants, with an incidence of 0.3–3.9%, often cooccurring with plagiocephaly and hip dysplasia. Early, targeted physiotherapy is the cornerstone of treatment, as it is crucial for preventing long-term deformities and promoting normal motor development. Current clinical practice guidelines for CMT emphasize early physiotherapy, primarily passive stretching and active range-of-motion exercises, which have the strongest evidence (Grade B recommendation) [1,2]. Neurodevelopmental approaches—Vojta Therapy and the NDT Bobath Concept—are widely used, particularly in Europe, despite limited high-quality comparative evidence. Both are resource-intensive, requiring specialized therapist training and substantial caregiver involvement, raising concerns about access equity and cost-effectiveness. Given their widespread use and substantial resource demands, rigorous comparative research is clinically and ethically important to inform evidence-based practice and optimize outcomes for vulnerable pediatric populations. Although both Vojta and Bobath approaches aim to normalize muscle tone through neuromotor pattern activation, their mechanisms differ. Vojta therapy leverages the systemic activation of innate reflex locomotion patterns to engage central motor circuits, while Bobath focuses on manual facilitation and inhibition techniques to modulate tone through peripheral sensory input. Evidence from related pediatric conditions suggests potential differences in postural symmetry and functional outcomes but CMT-specific comparative data are sparse and methodologically heterogeneous [3,4]. Comparative analyses are essential to determine which approach offers superior outcomes for specific pathologies like CMT [3,4,5,6].

Acquired torticollis, regardless of age, is regarded as a symptom of another underlying condition. Therefore, early clinical assessment and differential diagnosis of an infant are essential to identify the cause and implement appropriate treatment [6,7,8,9,10].

The primary treatment for congenital muscular torticollis is physiotherapy. In rare cases with significant mobility restrictions or failed conservative treatment, surgery is performed. However, in most cases, rehabilitation is sufficient for full recovery.

Treatment typically includes a comprehensive physiotherapy program involving stretching of neck muscles, strengthening of neck and trunk muscles, promoting symmetrical movements, environmental adaptation and caregiver education. Caregivers play a crucial role in implementing treatment recommendations on a daily basis. Physiotherapists should conduct sessions 1–2 times per week and teach parents how to perform exercises correctly at home.

Core elements of physiotherapy include:

- Increasing passive range of motion in the neck
- Increasing active range of motion in the neck and trunk
- Developing symmetrical, active movements of the whole body
- Environmental adaptation and parental education on appropriate child care—proper lifting of the child, carrying, placing in the cot, placing the child for feeding in a position that supports symmetrical neck position [1,2]

The choice of additional physiotherapeutic methods should be made by a medical rehabilitation specialist and a physiotherapist. Scientific evidence for other conservative treatments for congenital muscular torticollis, such as kinesio taping, soft tissue mobilization or orthotic bracing, is inconclusive. Some studies even suggest these techniques may be ineffective (e.g., myokinetic stretching, orthoses, orthopedic collars). The use of an orthopedic collar for rigid torticollis is controversial, as its effect is to restrict movement, which may weaken the muscle on the opposite side and exacerbate muscle tone imbalance. Active stimulation and proper positioning are now preferred. American guidelines discourage spinal manipulation in the cervical area, as clinical studies do not confirm the effectiveness of this method and there is a high risk that it may lead to breathing difficulties and, in rare cases, even death [1,2,11,12,13].

Vojta therapy, also known as reflex locomotion therapy, is a neurorehabilitation concept developed by Professor Václav Vojta. It is based on the activation of innate, genetically programmed motor patterns—so-called reflex locomotion—which may be blocked due to central nervous system damage. This therapy involves stimulating specific trigger zones in established therapeutic positions (lying on the back, stomach, or side), leading to the reactivation of global motor patterns such as trunk stabilization and limb activation.

Unlike conventional kinesiotherapy, Vojta therapy works on the level of central motor control, fostering the reorganization of the central nervous system. As a result, it improves coordination, balance, and motor planning and induces positive changes in the functioning of the respiratory, digestive, and circulatory systems as well as in the emotional and communicative spheres. Due to the systemic nature of its impact, reactions in different body parts can be triggered within a single session, supporting sensorimotor integration and normalization of muscle tone.

Therapy effectiveness depends on an individualized exercise plan based on neurodevelopmental assessment and tailored to the patient’s age and severity of symptoms. The regularity of the exercises and the involvement of the family are also crucial—exercises should be performed at home 3–4 times a day and visits to the therapist usually take place 1–2 times a week. A number of long-term benefits of therapy are presented in the literature, including improved postural control, higher quality of motor patterns, reduced compensatory muscle tension and better integration of motor and cognitive functions. Although therapy does not always lead to full normalization of function, it significantly increases the child’s developmental potential and quality of life [14,15,16,17].

The NDT Bobath Concept (Neuro-Developmental Treatment) is an integrated therapeutic approach aimed at the rehabilitation of people with damage to the central nervous system, with particular emphasis on children with cerebral palsy. The main principles of this approach include modulating muscle tone to prevent spastic and hypotonic states and forming correct motor patterns by eliminating compensatory movement patterns. Systematic therapy prevents secondary musculoskeletal deformities.

Therapy is holistic in nature, focusing on the functional integration of movements in daily activities and thus fostering postural control and balance. Through manual techniques, such as facilitation or inhibition of selected movement patterns, the therapist stimulates neuroplasticity, thereby supporting reorganization and adaptation of the central nervous system structures [18,19,20,21,22].

### Study Rationale and Objective

Despite the widespread clinical use of both methods and the known benefits of early intervention in CMT, a clear comparative effectiveness is lacking. Given the limited evidence base, high resource requirements, and ethical imperative to optimize care for vulnerable pediatric populations, the aim of this pilot study was to compare the effectiveness of two neurodevelopmental approaches—Vojta Therapy and the NDT Bobath concept in the treatment of congenital muscular torticollis in infants.

## 2. Materials and Methods

### 2.1. Study Design and Participants

The retrospective cohort pilot study was conducted at the Department of Rehabilitation of Polish Mother’s Memorial Hospital—Research Institute in Łódź, spanning the years 2016–2024, with patient follow-up until the age of 12 months. The sample size (*n* = 53) was pragmatic, determined by available departmental records meeting inclusion criteria during the study period. Post hoc power analysis indicated 80% power to detect a 5° difference in neck rotation (α = 0.05, effect size d = 0.8). This modest sample size limits generalizability and statistical power; findings should be interpreted as hypothesis-generating and require confirmation in larger prospective studies. Inclusion criteria: infants under five months of age with ultrasound-confirmed CMT (sternocleidomastoid muscle involvement), absence of neurological or orthopedic comorbidities, and parental commitment to attend biweekly therapy sessions and perform daily home exercises. Exclusion criteria: non-muscular torticollis (e.g., neurological, skeletal, ocular causes), congenital anomalies affecting motor development, lack of parental consent, or families requesting a specific therapy method (to reduce selection bias). Sample recruitment was conducted by the lead physiotherapist and supervising physician. Informed consent was obtained from parents/legal guardians by the attending physician using a form adapted for pediatric research and approved by the Bioethics Committee of Polish Mother’s Memorial Hospital—Research Institute (approval no. KB-108/2023, 12 December 2023), compliant with national regulations for research involving minors. Group assignment was strictly non-random and driven by therapist availability to minimize delay in therapy initiation (time-to-treatment). This allocation process, where infants were booked with the first available credentialed therapist, introduces potential selection bias and confounding factors (e.g., therapist-specific experience or scheduling artifacts), which are acknowledged as primary methodological limitations. All therapists were certified in their respective methods (Vojta or NDT Bobath) with ≥5 years of clinical experience in pediatric rehabilitation. Families requesting a specific method were excluded, and any parental request for a specific method was documented. The study pathway prioritized time-to-therapy start over waiting for a particular technique. Baseline characteristics (Table 1) were similar between groups, but unmeasured confounders (e.g., therapist experience variability, family socioeconomic factors) cannot be ruled out. For ethical reasons, no untreated control group (i.e., infants with CMT without any physiotherapeutic intervention) was included, as withholding standard care would contravene current clinical practice guidelines.

### 2.2. Intervention Protocols

Vojta therapy: two weekly, 30 min sessions for twenty weeks, targeting standardized reflex zones per Vojta protocol. NDT Bobath: two weekly, 30 min sessions for twenty weeks, focusing on facilitation techniques per NDT Bobath protocol. Caregivers were trained to perform prescribed exercises at home 3–4 times daily. Training consisted of standardized verbal and written instructions with demonstration and supervised practice during the first two therapy sessions. Instructions were reviewed and reinforced at each subsequent visit. The frequency of prescribed home exercises (3–4 times daily) was monitored through weekly verbal reports from caregivers, which were documented in the patient files by the treating physiotherapist. However, this method is subject to social desirability and recall bias; future studies should employ objective adherence measures (e.g., video logs, wearable device tracking). Compliance with the in-clinic session schedule (two weekly sessions) was tracked via appointment records. Only infants with documented 80% attendance for in-clinic sessions were included in the final analysis to ensure acceptable intervention fidelity.

### 2.3. Outcome Measures

Head tilt and neck rotation measured by two blinded doctors; interrater reliability ICC = 0.92. Measurements were performed using standardized goniometry at baseline and post-treatment (20 weeks). Functional outcomes (e.g., motor milestones, quality of movement) were not assessed.

#### 2.3.1. Ethics

The study complies with the Declaration of Helsinki and was approved by the Bioethics Committee of Polish Mother’s Memorial Hospital—Research Institute (approval no. KB-108/2023, approval date: 12 December 2023). Informed consent was obtained from parents/legal guardians using a form adapted for research involving minors.

#### 2.3.2. Statistical Analysis

Normally distributed continuous variables were described with mean and standard deviation (SD). For continuous variables not following normal distribution, median and interquartile range (IQR) were reported. Categorical variables were presented as counts with corresponding relative frequencies. Normality of continuous variables was assessed using Shapiro–Wilk test, skewness and kurtosis. Levene’s test was utilized to examine homogeneity of variance. Group comparisons were conducted using Student’s *t*-test, Mann–Whitney U test and Pearson’s chi-square test, as appropriate. Benjamini–Hochberg correction was applied to the comparisons to account for multiple testing. Outcome was considered significant if *p* < 0.05. All statistical analyses were performed using R software (R 4.1.2). To evaluate whether treatment effects differed by age at initiation, ANCOVA models were fitted for head tilt and neck rotation outcomes, adjusting for baseline values and including the interaction of therapy type (Vojta vs. NDT Bobath) with age group (1–2 vs. 3–4 months). Estimated marginal means with 95% confidence intervals were reported, with multiple comparisons corrected using the Benjamini–Hochberg procedure. Effect sizes were expressed as partial eta-squared and Hedges’ g for between-group comparisons. ANCOVA and marginal means were estimated using the car and emmeans packages in R.

No artificial intelligence tools were used in the preparation of this manuscript. All statistical analyses were performed using R software (R 4.1.2) without the use of artificial intelligence tools. Data interpretation, manuscript writing, and figure generation were conducted exclusively by the authors.

## 3. Results

### 3.1. Study Group Characteristics

The study sample consisted of *n* = 53 children with torticollis, aged from 1 month to 4 months. Mean age was 2.51 ± 0.87 months. The proportion of females was 45.3%. Over half of the group was born in natural birth (58.5%), while 41.5% was born through cesarean section. First birth was 52.8% of cases, second was 39.6% of cases, third was observed in *n* = 4 (7.5%). Right side of torticollis was observed slightly more frequently (54.7%). NDT Bobath was used in case of 45.3% of children, while the rest of the group went through Vojta (54.7%).

Participants split between therapy type groups were compared in terms of baseline characteristics, no significant difference between groups was revealed, Table 1.

### 3.2. Evaluation of Torticollis

**Figure 1:**
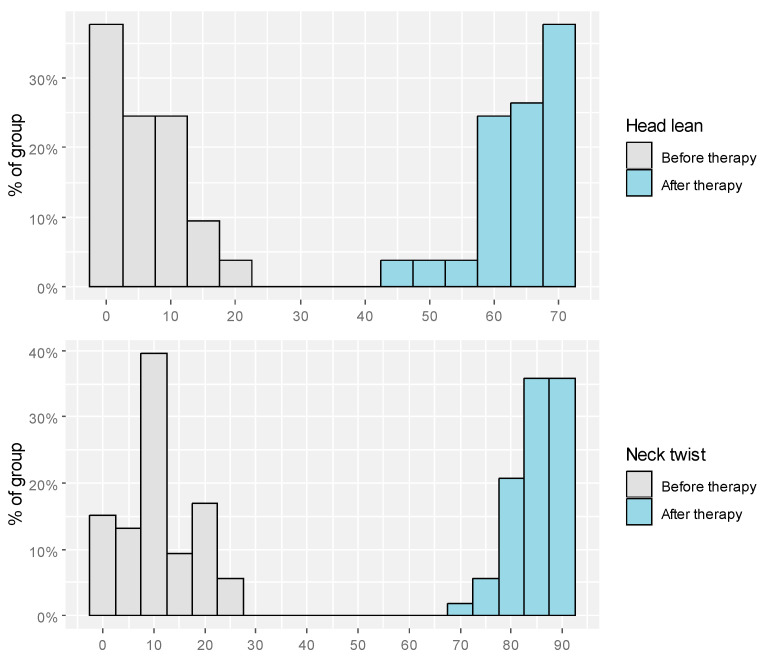
Distribution of torticollis measurements before and after therapy.

Head tilt before therapy ranged from 0.00 to 70.00, neck rotation before therapy ranged from 0.00 to 25.00. After the therapy, values for both measures fell within 45.00 to 70.00 and 70.00 to 90.00, respectively. The maximum values of head tilt and neck rotation after therapy were accomplished by 37.7% and 35.8%, respectively. Detailed descriptive statistics were presented in Table 2. Distribution of head tilt and neck rotation before and after therapy was presented as Figure 1.

### 3.3. Dependency Between Therapy Effectiveness and Selected Factors (Sex, Age and Type of Therapy)

Sex did not differentiate therapy effectiveness (*p* > 0.05), considering raw *p* values and *p* values after the correction for multiple testing Table 3.

Head tilt was significantly higher when therapy started at age 1–2 months compared to 3–4 months with difference in MD = 10.00 [5.00; 10.00], *p* < 0.001. Neck rotation was significantly higher when therapy started at age 1–2 months compared to 3–4 months with difference in MD = 7.50 [5.00; 10.00], *p* < 0.001. Proportion of patients with head tilt equal to 70 after therapy was 5× higher among children who started therapy at age of 1–2 months compared to 3–4 months, RR = 5.46 [1.81; 16.44], *p* < 0.001. Proportion of patients with neck rotation equal to 90 after therapy was 8x higher among children who started therapy at age of 1–2 months compared to 3–4 months, RR = 8.19 [2.10; 31.97], *p* < 0.001, After adjustment for multiple comparisons, the observed associations remained statistically significant, Table 4.

Neck rotation after therapy was significantly lower among children with NDT Bobath compared to children with Vojta with difference in MD = −2.87 [−5.52; −0.22], *p* = 0.034. Improvement in head tilt was significantly lower among children with NDT Bobath compared to children with Vojta with difference in MD = −5.69 [−8.13; −3.25], *p* < 0.001 (Hedges’g =1.29). Improvement in neck rotation was significantly lower among children with NDT Bobath compared to children with Vojta with difference in MD = −5.89 [−8.57; −3.21], *p* < 0.001 (Hedges’g = 1.21), Table 5.

**Figure 2:**
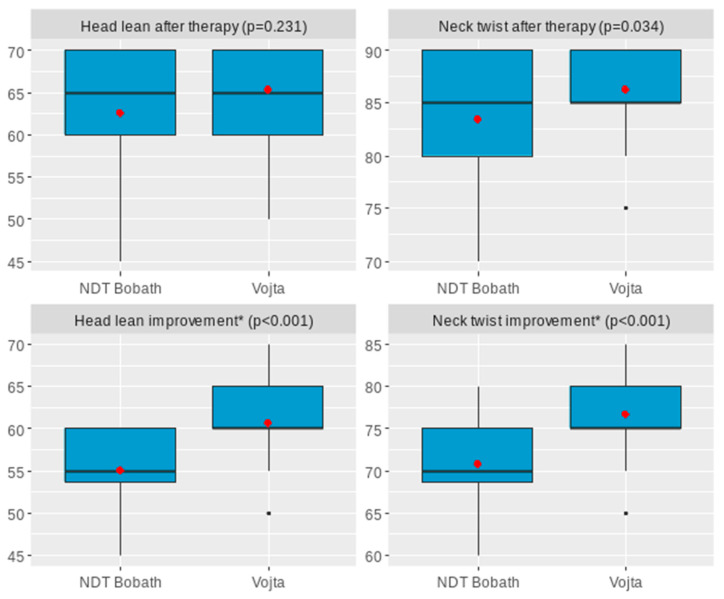
Boxplots presenting difference in therapy effectiveness by therapy type (thick line represents median, red dot represents mean, * change after therapy vs. before therapy).

Adjustment for multiple comparisons confirmed the statistical significance of all associations except for neck twist after therapy, which did not retain significance after correction (*p* adj = 0.068). Distribution of analyzed effectiveness measures split between therapy types was presented in Figure 2.

### 3.4. Lateralization of Torticollis in Relation to Sex

Proportion of left/right torticollis was not differentiated by sex (*p* = 0.726), Table 6.

### 3.5. Adjusted Comparative Analysis (ANCOVA with Interaction)

**Figure 3:**
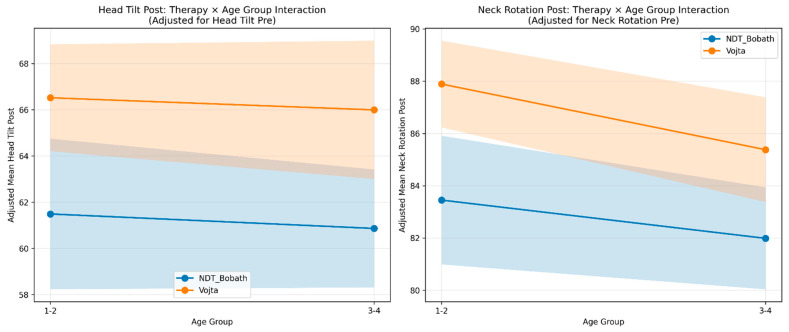
Therapy × treatment-initiation age group interaction (ANCOVA-adjusted means).

Table 7 presents the distribution of participants across the four study cells (*n* = 53). Based on this design, we calculated adjusted marginal means (EMMeans) and 95% CIs for post-treatment head tilt and neck rotation, controlling for baseline values. As shown in Figure 3, the outcome lines for Vojta therapy appear consistently higher than those for NDT Bobath across both age groups, suggesting a similar effect regardless of age.

Formal testing confirmed this visual impression. The Type-III ANCOVA detected no significant therapy × age interaction for either head tilt (Table 8) or neck rotation (Table 9). Consequently, we focused on the main effects. The detailed adjusted mean values, estimated at the average baseline severity, are provided in Table 10 for head tilt and Table 11 for neck rotation.

Distribution of participants across the four cells used in ANCOVA/ANOVA. Moderate balancing supports estimation of main effects, while limiting precision for the interaction.

Model specification: head_tilt_post ~ head_tilt_pre + therapy + age_group + therapy × age_group. Two-sided α = 0.05. Omnibus tests shown without multiplicity adjustment (see Methods).

Model specification: neck_rotation_post ~ neck_rotation_pre + therapy + age_group + therapy × age_group. Two-sided α = 0.05. Omnibus tests shown without multiplicity adjustment (see Methods).

Estimated at the mean of baseline head tilt. Higher values indicate performance closer to the 70° clinical target.

Estimated at the mean of baseline neck rotation. Higher values indicate performance closer to the 90° clinical target.

### 3.6. Interpretation Note

These results demonstrate statistically significant associations favoring Vojta therapy and early treatment initiation. However, due to the retrospective design, non-random allocation, and small sample size, causality cannot be established. The observed differences may reflect therapy effects, timing effects, or unmeasured confounders (e.g., baseline severity, therapist variability, family engagement). ANCOVA showed no therapy × age interaction, indicating that Vojta’s relative advantage over NDT Bobath was consistent across both age strata. Nonetheless, absolute outcomes were better when therapy was initiated earlier (1–2 months), suggesting independent benefits of early intervention. Additionally, outcomes were measured as angular improvements; functional outcomes (e.g., motor milestones, quality of movement, caregiver burden) were not assessed, limiting clinical interpretation.

## 4. Discussion

A comparative analysis of the effectiveness of the NDT Bobath and Vojta therapies in infants with congenital muscular torticollis (CMT) under 12 months of age revealed significant differences in the efficacy of both approaches. Our study (*n* = 53) demonstrated that Vojta therapy was associated with greater improvements in head position (MD = −5.69 [−8.13; −3.25], *p* < 0.001) and increasing the range of neck rotation (MD = −5.89 [−8.57; −3.21], *p* < 0.001) compared to NDT Bobath. However, the statistical significance of neck rotation improvement in the NDT Bobath group was not retained after adjustment for multiple comparisons (*p*_adj = 0.068), suggesting a need for cautious interpretation of this outcome. The medium-to-large effect sizes (Hedges’ g = 1.29 for head tilt; 1.21 for neck rotation) suggest that the observed angular differences may be clinically meaningful. These angular improvements brought infants closer to the defined clinical thresholds (70° head tilt, 90° neck rotation). However, we did not assess functional motor development, secondary complications, or quality of life; therefore, we cannot determine whether these angular improvements translate into functional benefits or improved long-term outcomes.

In the adjusted comparative analysis, ANCOVA revealed no evidence of a therapy-by-age interaction for either endpoint, indicating that the relative advantage of Vojta therapy over NDT Bobath was consistent across infants who started at 1–2 vs. 3–4 months (head tilt: *p* = 0.967; neck rotation: *p* = 0.582). After adjusting for baseline, the main effect of therapy favored Vojta for both head tilt (*p* = 0.009) and neck rotation (*p* = 0.003), whereas the main effect of treatment-initiation age did not reach statistical significance. Estimated marginal means corroborated these findings, with higher post-treatment values after Vojta in both age strata. These findings suggest two independent effects: (1) Vojta yields greater improvements than NDT Bobath at any given initiation age (therapy-specific effect), and (2) earlier treatment initiation (1–2 months) benefits both therapies (timing-specific effect). However, causality cannot be established due to the retrospective, non-randomized design. Observed associations may reflect unmeasured confounders such as baseline severity (not systematically quantified), degree of muscle fibrosis, therapist variability, or family engagement and adherence—factors that could independently influence outcomes. Clinically, when available, Vojta may be preferred without the need to delay treatment to “fit” a specific age band. The “early start” signal seen in unadjusted summaries (higher proportions reaching 70°/90° at 1–2 months) attenuated after baseline adjustment in continuous models, suggesting that baseline severity and/or regression to the mean account for part of this effect. This underscores the importance of baseline correction in observational studies and motivates prospective RCTs. Strong baseline–outcome relationships reinforce the need for rigorous baseline measurement/reporting. Future studies should consider longer follow-up and a combined-therapy arm (Vojta + NDT) to test synergy.

### 4.1. Quantitative Comparison with Previous Studies

Our findings align with and extend previous research on Vojta therapy and NDT Bobath in pediatric musculoskeletal conditions. Öhman et al. (2010) conducted a randomized pilot study (*n* = 59) comparing therapist-delivered versus parent-delivered stretching treatment in infants with CMT and reported significant improvements in cervical rotation and lateral flexion in both groups, with no significant difference between delivery methods after 6 months [14]. Our results showed similar or slightly smaller improvements (MD = −5.69° for head tilt, MD = −5.89° for neck rotation) after 20 weeks, which may reflect differences in baseline severity, intervention intensity, or measurement timing. Direct comparison is limited by methodological differences: Öhman et al. employed a stretching-focused protocol without distinguishing between Vojta and Bobath approaches, whereas our study directly compared two neurodevelopmental therapies with distinct mechanisms.

Castilla et al. (2023), in a systematic review (15 studies) informing the APTA Clinical Practice Guideline update for CMT, reported that active range-of-motion exercises and stretching programs improved cervical rotation by approximately 10–15° after 8–12 weeks, with very low to low certainty of evidence due to heterogeneity in intervention protocols and outcome measures [15]. Our NDT Bobath group showed more modest improvements (mean improvement ~4–5°), possibly reflecting differences in intervention intensity (2 sessions/week for 20 weeks in our study vs. more intensive protocols in Gupta et al.) or baseline characteristics. Notably, our Vojta group achieved improvements (~10°) comparable to the upper range reported in Castilla et al.’s systematic review, suggesting potential mechanistic advantages of reflex-based approaches over conventional stretching alone.

Jung et al. (2017) compared Vojta and NDT Bobath in a small RCT (*n* = 37) of infants with postural asymmetry and reported no significant difference between groups, though both improved [16]. This contrasts with our findings of Vojta superiority. Potential explanations include: (1) differences in condition severity (postural asymmetry vs. confirmed CMT), (2) outcome measures (our study used angular measures; Jung et al. used functional scales), (3) sample size and power (both studies were underpowered for equivalence testing), and (4) therapist variability. Jung et al.’s findings of comparable parent compliance between Vojta (98.6%) and NDT (97.3%) suggest that differences in home exercise adherence are unlikely to explain our observed superiority of Vojta. These discrepancies underscore the need for larger, adequately powered RCTs with standardized protocols and outcome measures to clarify comparative efficacy. Recent systematic evidence from Antares et al. (2025) supports our findings, demonstrating that adding manual therapy to active control in CMT resulted in short-term improvements in passive cervical rotation (OR 9.79, 95%CI 4.26–22.50), passive cervical lateroflexion (OR 2.66, 95%CI 1.17–6.04), and symmetric head posture (OR 4.55, 95%CI 2.57–8.05) [7]. However, certainty of evidence remains very low to low due to high risk of bias, small sample sizes, and study heterogeneity—limitations that our study also shares. Antares et al. emphasized the urgent need for large definitive trials with standardized outcome measures, a recommendation directly aligned with our study’s conclusions.

Overall, our results are consistent with the literature suggesting that neurodevelopmental therapies (Vojta, NDT Bobath) can improve cervical mobility in CMT, with Vojta potentially offering advantages in angular improvement. However, heterogeneity in study design, intervention protocols, and outcome measures limits definitive conclusions and highlights the need for standardized comparative research.

### 4.2. Methodological Limitations

A key methodological limitation of our study is its retrospective, non-randomized design, which introduces a high potential for selection bias. Specifically, the allocation based on therapist availability and scheduling is a significant confounding factor, as it means the groups were not truly balanced, and differences in therapist experience or patient-therapist rapport may have influenced the outcomes. Although baseline characteristics were similar (Table 1), unmeasured variables (e.g., therapist expertise variability, family socioeconomic status, caregiver motivation) cannot be excluded. Baseline severity was not systematically quantified (e.g., degree of muscle fibrosis, sternocleidomastoid thickness), which may confound therapy response. The relatively small sample size (*n* = 53) further limits the statistical power, making it difficult to detect smaller, yet potentially clinically relevant, differences, and significantly restricts the generalizability of the findings to the broader CMT population. This constraint is consistent with other studies in the field of pediatric rehabilitation for CMT. For instance, comparable trials by Jung et al. (2017, *n* = 37) and and Öhman et al. (2010, *n* = 59) highlight that small sample sizes are common in this research area due to the specific nature of the condition and challenges in recruiting large cohorts of infants [14,16]. Adherence to home exercises was monitored via caregiver-completed logs, which are subject to social desirability and recall bias; objective adherence measures (e.g., video logs, smart device tracking) are needed in future studies. We did not assess functional outcomes (motor milestones, quality of movement, caregiver burden) or long-term follow-up beyond 12 months, limiting clinical applicability. Finally, both Vojta and NDT Bobath have limited and methodologically heterogeneous evidence bases in CMT; current guidelines emphasize passive stretching and active range-of-motion exercises (Grade B), while neurodevelopmental approaches are widely used but lack high-level comparative evidence [8,9]. This common limitation must be addressed by large-scale, multi-center, and preferably prospective studies.

### 4.3. Therapist-Related Confounding

A critical limitation of our study is the potential for therapist-related confounding. Although all therapists were certified in their respective methods (Vojta or NDT Bobath) with ≥5 years of clinical experience in pediatric rehabilitation, we did not systematically assess or control for several therapist-level factors that may influence outcomes:

1. Individual therapist skill and expertise variability: Within each therapy modality, individual therapists may differ in technical proficiency, clinical reasoning, and ability to adapt interventions to individual patient needs. These differences could introduce outcome variability independent of the therapy type itself.
2. Intervention fidelity: We did not employ structured fidelity monitoring (e.g., session video review, protocol checklists) to ensure consistent delivery of Vojta and NDT Bobath protocols across therapists and sessions. Deviations from standardized protocols may have occurred, potentially affecting comparability.
3. Therapeutic alliance and family engagement: Therapist communication style, empathy, and ability to engage and motivate caregivers may significantly impact adherence to home exercises and, consequently, outcomes. This “therapist effect” is well-documented in rehabilitation research but was not measured in our study.
4. Allocation mechanism: Because group assignment was determined by therapist availability rather than randomization, systematic differences in therapist characteristics between the Vojta and NDT Bobath groups cannot be excluded. For example, if more experienced or skilled therapists were systematically more available for one therapy type, this could bias results in favor of that therapy.

These therapist-related factors may account for part of the observed difference between Vojta and NDT Bobath, and it is difficult to disentangle therapy-specific effects from therapist-specific effects in our retrospective design. Future studies should employ randomization, multi-therapist designs (with therapists delivering both interventions to balance therapist effects), intervention fidelity monitoring, and statistical adjustment for therapist clustering (e.g., mixed-effects models with therapist as a random effect) to isolate therapy-specific effects and improve internal validity.

### 4.4. Long-Term Implications and Functional Outcomes

Although our study demonstrated statistically significant and clinically meaningful improvements in head tilt and neck rotation—bringing infants closer to functional thresholds (70° head tilt, 90° neck rotation)—we did not assess broader functional developmental outcomes or long-term implications. This represents a key limitation, as the ultimate goal of CMT treatment is not merely to improve angular measurements but to optimize functional motor development, prevent secondary complications, and enhance quality of life.

Potential long-term implications of improved cervical mobility include:

1. Motor milestone attainment: Persistent head tilt and limited cervical rotation may delay motor milestones such as rolling, sitting, crawling, and walking by affecting postural control, weight shifting, and visual-spatial orientation. Early correction of CMT may facilitate timely milestone achievement, though this hypothesis requires longitudinal assessment with standardized developmental scales (e.g., Alberta Infant Motor Scale, Bayley Scales of Infant Development).
2. Postural symmetry and craniofacial development: Untreated or inadequately treated CMT is associated with persistent postural asymmetry, plagiocephaly, facial asymmetry, and scoliosis [5,6]. Improved cervical mobility may reduce these risks by promoting symmetrical head positioning, balanced muscle development, and normalized biomechanical loading. Long-term follow-up (≥24 months) with postural assessment and craniofacial imaging would clarify whether early angular improvements translate into sustained postural symmetry.
3. Quality of life and caregiver burden: CMT may impact infant comfort, feeding, sleep, and social interaction (e.g., limited visual engagement due to restricted head turning). Effective treatment may enhance infant well-being and reduce caregiver stress and burden. Future studies should incorporate caregiver-reported outcome measures (e.g., Pediatric Quality of Life Inventory, Infant Comfort Scale) to capture these dimensions.
4. Prevention of surgical intervention: Approximately 5–10% of CMT cases require surgical release of the sternocleidomastoid muscle when conservative treatment fails [8]. Effective early physiotherapy may reduce the need for surgery, with associated benefits in cost, risk, and recovery time. Comparative studies should assess surgery rates as a long-term outcome.

Our study’s 12-month follow-up and focus on angular measurements does not address these important outcomes. Future research should employ comprehensive, multidimensional outcome batteries including standardized developmental assessments, functional movement quality scales, craniofacial symmetry measures, caregiver-reported quality of life and burden scales, and long-term follow-up (≥24 months) to assess durability and functional integration of improvements. Such comprehensive assessment will clarify whether the angular improvements observed in our study—and the apparent advantage of Vojta therapy—translate into meaningful long-term functional, developmental, and quality-of-life benefits.

### 4.5. Clinical and Ethical Context

Both Vojta and NDT Bobath are resource-intensive therapies requiring specialized training and substantial caregiver involvement, raising concerns about access equity and cost-effectiveness. Given their widespread clinical use despite limited evidence, comparative research is clinically and ethically important to inform practice and optimize resource allocation.

Our findings align with the latest guidelines from the APTA Academy of Pediatric Physical Therapy, which recommend early physical therapy (including both Vojta and NDT Bobath approaches) for CMT, noting the potential for rapid functional improvement in postural control [1,2]. Sargent et al. (2019) similarly highlighted the efficacy of Vojta therapy in restoring symmetry and mobility in infants with CMT, emphasizing the importance of early intervention [1]. Our results further support this, showing that initiating therapy at 1–2 months of age significantly improved head tilt (MD = 10.00 [5.00; 10.00], *p* < 0.001) and neck rotation (MD = 7.50 [5.00; 10.00], *p* < 0.001) compared to initiation at 3–4 months. Notably, the proportion of patients achieving optimal head tilt (70°) and neck rotation (90°) was 5 and 8 times higher, respectively, in the early intervention group (RR = 5.46 [1.81; 16.44], *p* < 0.001; RR = 8.19 [2.10; 31.97], *p* < 0.001) [23]. These outcomes were consistent across both therapy groups, though the Vojta group demonstrated greater improvements in both measures. This suggests that while early intervention is critical, Vojta therapy was associated with greater angular improvement, though this represents an association rather than a causal relationship, and the clinical significance requires validation through functional outcome assessment.

The mechanistic differences between the two therapies, as discussed in Section 4.2, may explain the observed differences in efficacy. Recent systematic evidence from Sánchez-González et al. (2024) confirms the neurophysiological robustness of Vojta therapy’s reflex locomotion mechanisms, while Qian et al. (2025) demonstrated immediate biomechanical effects on postural control [24,25]. Conversely, Acar et al. (2022) and Besios et al. (2018) highlight the versatility of the NDT Bobath approach in addressing sensory, feeding, and autonomic functions, suggesting its broader applicability in complex cases [19,21]. Antares et al. (2025), in their systematic review of 100 studies (*n* = 8125), further confirmed the efficacy of manual therapy interventions in improving cervical rotation and symmetric head posture in CMT, though they noted that certainty of evidence remains very low to low due to methodological heterogeneity [7]. These findings collectively underscore the importance of tailoring therapy to the developmental stage and specific needs of the infant—as emphasized by Obajeun et al. (2023)—particularly when structural changes or comorbidities are present [9]. However, the critical knowledge gap identified by Antares et al. (2025) [7]—the lack of large, adequately powered RCTs with standardized protocols—must be addressed to move beyond associations and establish causality.

### 4.6. Future Research Directions

Prospective randomized controlled trials with larger, diverse cohorts are essential to confirm these findings and establish causality. Studies should: (1) systematically quantify baseline severity (e.g., ultrasound-based muscle thickness, fibrosis grading), (2) employ objective adherence monitoring (video logs, wearable devices), (3) assess functional outcomes (motor milestones, quality of movement, caregiver burden, quality of life), (4) include longer follow-up (≥24 months) to evaluate durability, (5) explore combined Vojta–NDT Bobath protocols to test synergistic effects, and (6) employ multi-therapist designs with intervention fidelity monitoring and statistical adjustment for therapist clustering. Addressing these methodological gaps will strengthen evidence for optimal CMT management and inform clinical practice guidelines.

## 5. Conclusions

This retrospective pilot study demonstrates that Vojta therapy was associated with greater angular improvements in head tilt and cervical rotation compared to NDT Bobath in infants with CMT (*n* = 53), with large effect sizes (Hedges’ g > 1.2). Early intervention (1–2 months) was associated with better angular outcomes. However, only biomechanical (angular) outcomes were measured; functional outcomes (motor milestones, quality of movement, complications prevention, quality of life) were not assessed. The retrospective, non-randomized design introduces selection bias and precludes causal inference. Observed associations may reflect therapy effects, timing effects, therapist variability, or unmeasured confounders. Within these limitations, the findings suggest potential clinical relevance warranting further investigation. Prospective randomized controlled trials with larger samples, functional outcome measures, objective adherence monitoring, and long-term follow-up (≥24 months) are needed to confirm these associations, establish causality, and determine whether angular improvements translate into meaningful functional benefits.

## Figures and Tables

**Table 1:** Study group characteristics (*n* = 53).

Variable	Total Group	NDT Bobath	Vojta	MD/RR (95% CI)	*p*
N	53	24	29	-	-
Sex, female, *n* (%)	24 (45.3)	11 (45.8)	13 (44.8)	1.02 (0.56; 1.85)	>0.999
Age, months, mean ± SD	2.51 ± 0.87	2.62 ± 0.77	2.41 ± 0.95	0.21 (−0.27; 0.69)	0.383
Age, months, *n* (%)					
1	6 (11.3)	1 (4.2)	5 (17.2)	-	0.535
2	21 (39.6)	10 (41.7)	11 (37.9)		
3	19 (35.8)	10 (41.7)	9 (31.0)		
4	7 (13.2)	3 (12.5)	4 (13.8)		
Delivery type, *n* (%)					
Natural	31 (58.5)	14 (58.3)	17 (58.6)	-	>0.999
Cesarean section	22 (41.5)	10 (41.7)	12 (41.4)		
Birth, *n* (%)					
1st	28 (52.8)	11 (45.8)	17 (58.6)	-	0.641
2nd	21 (39.6)	11 (45.8)	10 (34.5)		
3rd	4 (7.5)	2 (8.3)	2 (6.9)		
Side of torticollis, *n* (%)					
Left	24 (45.3)	13 (54.2)	11 (37.9)	-	0.366
Right	29 (54.7)	11 (45.8)	18 (62.1)		
Head tilt before therapy, mean ± SD	5.85 ± 5.78	7.50 ± 6.43	4.48 ± 4.88	3.02 (−0.10; 6.14)	0.058
Neck rotation before therapy, median (IQR)	10.00 (5.00; 15.00)	10.00 (8.75; 20.00)	10.00 (5.00; 10.00)	0.00 (0.00; 10.00)	0.178

SD—standard deviation. IQR—interquartile range, MD—mean or median difference (NDT Bobath vs. Vojta), RR—relative risk (NDT Bobath vs. Vojta). Groups compared with *t*-Student test, Mann–Whitney U test, Pearson’s chi-square test or Fisher’s exact test, as appropriate.

**Table 2:** Evaluation of torticollis before and after the therapy.

Variable	Mean ± SD/*n* (%)	Median (IQR)	Range
Before therapy			
Head tilt	5.85 ± 5.78	5.00 (0.00; 10.00)	0.00–20.00
Neck rotation	10.85 ± 7.12	10.00 (5.00; 15.00)	0.00–25.00
After therapy			
Head tilt	63.96 ± 6.53	65.00 (60.00; 70.00)	45.00–70.00
Neck rotation	84.91 ± 4.95	85.00 (80.00; 90.00)	70.00–90.00
Head tilt maximum (70)	20 (37.7)	-	-
Neck rotation maximum (90)	19 (35.8)	-	-
Improvement (change after therapy vs. before therapy)			
Head tilt	58.11 ± 5.21	60.00 (55.00; 60.00)	45.00–70.00
Neck rotation	74.06 ± 5.64	75.00 (70.00; 80.00)	60.00–85.00

SD—standard deviation, IQR—interquartile range.

**Table 3:** Dependency between therapy effectiveness and sex.

Variable	Females	Males	MD/RR (95% CI)	*p*	*p* adj
Head tilt after therapy, median (IQR)	65.00 (60.00; 70.00)	65.00 (60.00; 70.00)	0.00 (−5.00; 0.00)	0.681	>0.999
Neck rotation after therapy, median (IQR)	85.00 (83.75; 90.00)	85.00 (80.00; 90.00)	0.00 (0.00; 5.00)	0.756	>0.999
Head tilt maximum (70) after therapy, *n* (%)	9 (37.5)	11 (37.9)	0.99 (0.49; 1.98)	>0.999	>0.999
Neck rotation maximum (90) after therapy, *n* (%)	9 (37.5)	10 (34.5)	1.09 (0.53; 2.23)	>0.999	>0.999
Head tilt improvement, median (IQR) *	60.00 (55.00; 60.00)	60.00 (55.00; 60.00)	0.00 (−5.00; 0.00)	0.546	>0.999
Neck rotation improvement, mean ± SD *	74.38 ± 5.95	73.79 ± 5.45	0.58 (−2.57; 3.73)	0.712	>0.999

SD—standard deviation, IQR—interquartile range, MD—mean or median difference (females vs. males), RR—relative risk (females vs. males). Groups compared with *t*-Student test Mann–Whitney U test or Pearson’s chi-square test, as appropriate. *p* adj—initial *p* values after applying Benjamini–Hochberg correction. * Change after therapy vs. before therapy.

**Table 4:** Dependency between therapy effectiveness and age.

Variable	1–2 Months	3–4 Months	MD/RR (95% CI)	*p*	*p* adj
Head tilt after therapy, median (IQR)	70.00 (65.00; 70.00)	60.00 (60.00; 65.00)	10.00 (5.00; 10.00)	<0.001	<0.001
Neck rotation after therapy, median (IQR)	90.00 (85.00; 90.00)	82.50 (80.00; 85.00)	7.50 (5.00; 10.00)	<0.001	<0.001
Head tilt maximum (70) after therapy, *n* (%)	17 (63.0)	3 (11.5)	5.46 (1.81; 16.44)	<0.001	0.001
Neck rotation maximum (90) after therapy, *n* (%)	17 (63.0)	2 (7.7)	8.19 (2.10; 31.97)	<0.001	<0.001
Head tilt improvement, mean ± SD *	57.96 ± 4.22	58.27 ± 6.16	−0.31 (−3.21; 2.59)	0.833	0.833
Neck rotation improvement, mean ± SD *	72.78 ± 6.25	75.38 ± 4.67	−2.61 (−5.66; 0.45)	0.093	0.111

SD—standard deviation, IQR—interquartile range, MD—mean or median difference (1–2 months vs. 3–4 months), RR—relative risk (1–2 months vs. 3–4 months). Groups compared with *t*-Student test Mann-Whiney U test or Pearson’s chi-square test, as appropriate. *p* adj—initial *p* values after applying Benjamini–Hochberg correction. * Change after therapy vs. before therapy.

**Table 5:** Dependency between therapy effectiveness and type of therapy.

Variable	NDT Bobath	Vojta	MD/RR (95% CI)	*p*	*p* adj	Hedges’g
Head tilt after therapy, median (IQR)	65.00 (60.00; 70.00)	65.00 (60.00; 70.00)	0.00 (−5.00; 0.00)	0.231	0.347	
Neck rotation after therapy, median (IQR)	83.33 ± 5.65	86.21 ± 3.93	−2.87 (−5.52; −0.22)	0.034	0.068	0.59
Head tilt maximum (70) after therapy, *n* (%)	8 (33.3)	12 (41.4)	0.81 (0.40; 1.64)	0.751	0.751	
Neck rotation maximum (90) after therapy, *n* (%)	7 (29.2)	12 (41.4)	0.70 (0.33; 1.51)	0.525	0.630	
Head tilt improvement, mean ± SD *	55.00 ± 4.66	60.69 ± 4.17	−5.69 (−8.13; −3.25)	<0.001	<0.001	1.29
Neck rotation improvement, mean ± SD *	70.83 ± 5.04	76.72 ± 4.68	−5.89 (−8.57; −3.21)	<0.001	<0.001	1.21

SD—standard deviation, IQR—interquartile range, MD—mean or median difference (NDT Bobath vs. Vojta), RR—relative risk (females vs. males). Groups compared with *t*-Student test Mann–Whitney U test or Pearson’s chi-square test, as appropriate. *p* adj—initial *p* values after applying Benjamini–Hochberg correction. * Change after therapy vs. before therapy.

**Table 6:** Dependency between side of torticollis and sex.

Variable	Females	Males	*p*
Side of torticollis, *n* (%)			
Left	12 (50.0)	12 (41.4)	0.726
Right	12 (50.0)	17 (58.6)	

Groups compared with Pearson’s chi-square test.

**Table 7:** Cell sizes in the 2 × 2 design (therapy × age at treatment initiation).

Therapy	Age Group	*n*
NDT Bobath	1–2 mo	11
NDT Bobath	3–4 mo	13
Vojta	1–2 mo	16
Vojta	3–4 mo	13

**Table 8:** Type-III ANCOVA for post-treatment head tilt (adjusted for baseline head tilt).

Effect	F	*p*-Value	Partial η^2^
Intercept	634.44	<0.001	0.946
Therapy (Vojta vs. NDT Bobath)	7.43	0.009	0.011
Age group (1–2 vs. 3–4 months)	0.08	0.776	0.000
Therapy × age group	0.00	0.967	0.000
Baseline head tilt (covariate)	28.41	<0.001	0.042

**Table 9:** Type-III ANCOVA for post-treatment neck rotation (adjusted for baseline neck rotation).

Effect	F	*p*	Partial η^2^
Intercept	1641.73	<0.001	0.981
Therapy (Vojta vs. NDT Bobath)	9.81	0.003	0.006
Age group (1–2 vs. 3–4 months)	0.73	0.398	0.000
Therapy × age group	0.31	0.582	0.000
Baseline neck rotation (covariate)	21.48	<0.001	0.013

**Table 10:** ANCOVA-adjusted marginal means (EMMeans) for post-treatment head tilt (degrees).

Therapy	Age Group	Adjusted Mean	SE	95% CI (Lower; Upper)
NDT Bobath	1–2 mo	61.49	1.62	58.23; 64.75
NDT Bobath	3–4 mo	60.87	1.27	58.31; 63.42
Vojta	1–2 mo	66.52	1.15	64.21; 68.84
Vojta	3–4 mo	66.00	1.49	63.00; 68.99

**Table 11:** ANCOVA-adjusted marginal means (EMMeans) for post-treatment neck rotation (degrees).

Therapy	Age Group	Adjusted Mean	SE	95% CI (Lower; Upper)
NDT Bobath	1–2 mo	83.45	1.22	80.99; 85.91
NDT Bobath	3–4 mo	81.99	0.97	80.04; 83.94
Vojta	1–2 mo	87.89	0.83	86.23; 89.55
Vojta	3–4 mo	85.38	1.00	83.38; 87.38

## Data Availability

The raw data supporting the conclusions of this article will be made available by the authors on request.

## Abbreviations

The following abbreviations are used in this manuscript:

NDT	Neuro-Developmental Treatment
APTA	American Physical Therapy Association
